# Clinicopathological Features, Epidermal Growth Factor Receptor (EGFR) Mutations and Anaplastic Lymphoma Kinase (ALK) Rearrangement-Based Survival of Patients With Advanced Non-small Cell Lung Cancer in a Tertiary Care Setting

**DOI:** 10.7759/cureus.76257

**Published:** 2024-12-23

**Authors:** Sameen Bin Naeem, Zeeshan Tariq, Mansoor Abbas, Farhana Badar, Rameen Shahid, Sadia Hassan, Farah Asif

**Affiliations:** 1 Medical Oncology, Shaukat Khanum Memorial Cancer Hospital and Research Centre, Lahore, PAK; 2 Clinical Research, Shaukat Khanum Memorial Cancer Hospital and Research Centre, Lahore, PAK

**Keywords:** alk rearrangement, clinicopathological features, egfr mutations, metastatic non-small cell lung cancer, outcomes

## Abstract

Background

Lung cancer is the most frequent cause of cancer-related deaths and the most common type of cancer globally. It is generally classified into two main histologic subtypes: non-small cell lung cancer (NSCLC) and small cell lung cancer (SCLC). NSCLC is the most prevalent type and is enriched with genetic and molecular diversity. This study evaluated the clinical, molecular, and demographic characteristics of patients with NSCLC, with a focus on variables involving disease stage, survival rates, and mutations in the epidermal growth factor receptor (EGFR) and anaplastic lymphoma kinase (ALK) genes.

Methods

This retrospective study included 51 adult patients aged 30 years and older. Only patients who received treatment and subsequent follow-up at our institution were included in this study.

Results

There were 51 patients, aged 30-84 years (mean = 59.6 ± 10.9). Out of 51 patients, 32 (64.7%) were men; 19 (37.2.5%) were either current or former smokers; 34 patients (66.7%) had an Eastern Cooperative Oncology Group (ECOG) performance status of either 0 or 1; 47 (92.2) had an adenocarcinoma; 15 (29.4%) had a bilateral lung disease; 43 (84.3%) had stage IV disease; 10 (19.6%) had a positive EGFR status; eight (15.7) had a positive ALK status; and 38 patients (73.1%) had died by the cut-off date for this study. The median survival time for the EGFR-negative patients was 15 months, as opposed to 16 months for those who were EGFR-positive. Likewise, the median survival time for both the ALK-negative and positive patients was 17 months each.

Conclusion

The study contributes to our understanding of NSCLC and highlights the trends of our region while acknowledging the limitations associated with molecular studies and smaller sample sizes. These findings are not aligned with global trends in NSCLC due to the above-mentioned reasons. Future prospective trials are needed to aim for larger cohorts and consider additional variables to address the complexities of NSCLC.

## Introduction

Lung cancer is the most common cancer with an estimated 12.4% of total cancer cases. It is the most common cause of cancer-related death with an estimated 1.8 million deaths worldwide [1]. In Pakistan, there were an estimated 10,000 new cases accounting for 5.9% of total cancer patients in 2020, and it is the fourth most common cause of cancer-related mortality among both sexes in Pakistan [2]. Lung cancer is generally categorized into two main histologic types: non-small cell lung cancer (NSCLC) and small cell lung cancer (SCLC). NSCLC accounts for 85% of all lung cancer cases [2] and regardless of its histologic subtypes, NSCLC is one of the most genetically and molecularly diverse cancers, posing significant challenges to prevention and treatment approaches [3]. The paradigm for managing advanced-stage NSCLC has already shifted due to the introduction of new drug classes, even though the shift from empiric to mechanism-based, molecular biomarker-driven therapeutic decision-making is a continuous process [4]. The International Association for the Study of Lung Cancer, the College of American Pathologists, and the Association of Molecular Pathology released molecular testing guidelines in 2013 that limited testing to polymerase chain reaction (PCR) for epidermal growth factor receptor (EGFR; also known as ERBB1) mutations and fluorescence in situ hybridization (FISH) for anaplastic lymphoma kinase (ALK) fusions [5]. Advancements in DNA-based next-generation sequencing (NGS) have enabled the identification of numerous gene alterations within a single tissue sample. This, combined with the development of effective tyrosine kinase inhibitors (TKI), has uncovered additional actionable driver mutations, including ROS1 fusions (2%), BRAF mutations (2%), NTRK fusions (1%), EGFR and HER2 exon 20 insertion mutations (3%), MET amplifications or exon 14 skipping mutations (2%), RET proto-oncogene rearrangements (1%), and KRAS mutations (25%). These discoveries have broadened the understanding of molecular profiles in lung cancer [6]. The mainstay of personalized treatment is adapting cancer therapy for each patient by using targeted therapies based on specific genetic aberrations [7]. In Pakistan, the diagnosis of lung cancer is often delayed and poses significant challenges in timely intervention and treatment initiation. Factors contributing to delayed diagnosis include limited awareness, insufficient access to healthcare facilities, and socioeconomic barriers. The therapeutic and prognostic implications of delayed diagnosis are significant, as lung cancer is frequently diagnosed at advanced stages, limiting the efficacy of available treatment options.

## Materials and methods

Study design and participants

This is a retrospective, cross-sectional study to describe the patient demographics, prevalence of EGFR and ALK mutations, and pathologic features and to correlate these with treatment outcomes in patients with biopsy-proven NSCLC. All consecutive eligible patients diagnosed with NSCLC and treated at Shaukat Khanum Memorial Cancer Hospital and Research Centre (SKMCH&RC) between January 1, 2017, and December 31, 2021, were included in the present study. Initially, 553 patients diagnosed with NSCLC were identified, since the study aimed to assess survival outcomes in mutation-known patients, therefore, only 51 patients were found to meet the eligibility criteria. The eligible study population comprised both males and females aged 18 years or above, with histologically confirmed NSCLC and known EGFR or ALK mutation status, who received treatment at SKMCH&RC. Patients with mixed histology of SCLC and NSCLC histology and with insufficient available data were excluded from the analysis. The study was approved as a retrospective study by the Institutional Review Board (IRB) of SKMCH&RC and the IRB also granted a waiver of informed consent. Data used in the analysis was anonymized.

Methods

Information regarding the demographic distribution, baseline characteristics of the patients, as well as data for clinical and pathologic parameters were obtained from patients’ medical records present in the electronic hospital information system and were analyzed. The histological diagnosis for the absence or presence of the EGFR mutations and ALK rearrangement was confirmed by existing reports. The follow-up outcome assessed was overall survival. Follow-up data were collected from electronic patient records. Overall survival was assessed based on the interval duration between the date of diagnosis and the date of last follow-up and patient status at the last follow-up (alive, dead, unknown).

Statistical analysis

All enrolled patients with histologically confirmed NSCLC and with available mutation status information were included in the study. The counts and percentages were calculated for categorical variables. Descriptive statistics were generated for continuous variables. The counts were cross-tabulated between the final patient status and sex, smoking status, Eastern Cooperative Oncology Group (ECOG) performance status, laterality, morphology, disease stage, EGFR status, and ALK status. Accordingly, the chi-square statistics were computed. A logistic regression analysis was also conducted. The survival analysis was done through the Kaplan-Meier method, comparing the survival distribution by EGFR and ALK status. All results were considered statistically significant at an alpha level of 0.05.

## Results

There were 51 patients in the study, whose age of presentation ranged from 30 to 84 years (mean = 59.6 ± 10.9; mode = 62). Out of these 51 patients, 32 (64.7%) were men; 19 (37.2.5%) were either current or former smokers; 34 patients (66.7%) had an ECOG performance status of either 0 or 1; 47 (92.2%) had an adenocarcinoma; 43 (84.3%) had stage IV disease; 10 (19.6%) had a positive EGFR status; eight (15.7%) had a positive ALK status; and 38 patients (73.1%) had died by the cut-off date for this study. The cross-tabulation between the final patient status (dead versus alive) and various factors mentioned above did not show any significant associations (p-value > 0.05). The details are shown in Table 1.

**Table 1:** Demographic details and clinical features of the patients in the study. * The chi-square test and Fisher’s exact test have been applied. ** ECOG: Eastern Cooperative Oncology Group. ECOG performance status: 50% of the cells have an expected count of less than five. *** Laterality: 33.3% of the cells have an expected count of less than five. ∞ EGFR is an abbreviation for epidermal growth factor receptor. ~ ALK is an abbreviation for the anaplastic lymphoma kinase.

		Patient status, count (%)		Total count (%)	P-value*
Variable	Category	Alive, 13 (25.5)	Dead, 38 (74.5)	51 (100)	
Sex	Female	3 (16.7)	15 (83.3)	18 (35.3)	0.336
	Male	10 (30.3)	23(69.7)	33 (64.7)	
Smoking status	Current smoker	4 (40.0)	6 (60.0)	10 (19.6)	0.351
	Former smoker	1 (11.1)	8 (88.9)	9 (17.6)	
	Never smoker	8 (25.0)	24 (75.0)	32 (62.8)	
ECOG performance status**	Grade 0	5 (35.7)	9 (64.3)	14 (27.5)	0.149
	Grade I	7 (35)	13 (65)	20 (39.2)	
	Grade II	1 (9.1)	10 (90.9)	11 (21.5)	
	Grade III	0 (0.0)	6 (100)	6 (11.8)	
Laterality***	Right lung	4 (20.0)	16 (80.0)	20 (39.2)	0.669
	Left lung	4 (25.0)	12 (75.0)	16 (31.4)	
	Right and left lung	5 (33.3)	10 (66.7)	15 (29.4)	
Morphology	Adenocarcinoma	12 (25.5)	35 (74.5)	47 (92.2)	1
	Squamous cell carcinoma	1 (25.0)	3 (75.0)	4 (7.8)	
Disease stage	Stage III	3 (37.5)	5 (62.5)	8 (15.7)	0.404
	Stage IV	10 (23.3)	33 (76.7)	43 (84.3)	
EGFR∞	Negative	11 (27.5)	29 (72.5)	40 (78.4)	1
	Positive	2 (20.0)	8 (80.0)	10 (19.6)	
ALK~	Negative	9 (30.0)	21 (70.0)	30 (58.9)	0.689
	Positive	3 (37.5)	5 (62.5)	8 (15.7)	

**Figure 1:**
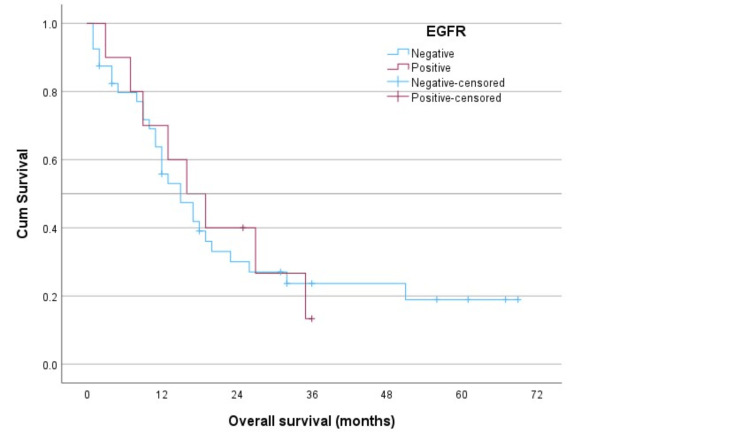
The survival distributions by the epidermal growth factor receptor (EGFR) status in lung cancer patients using the Kaplan-Meier analysis.

**Figure 2:**
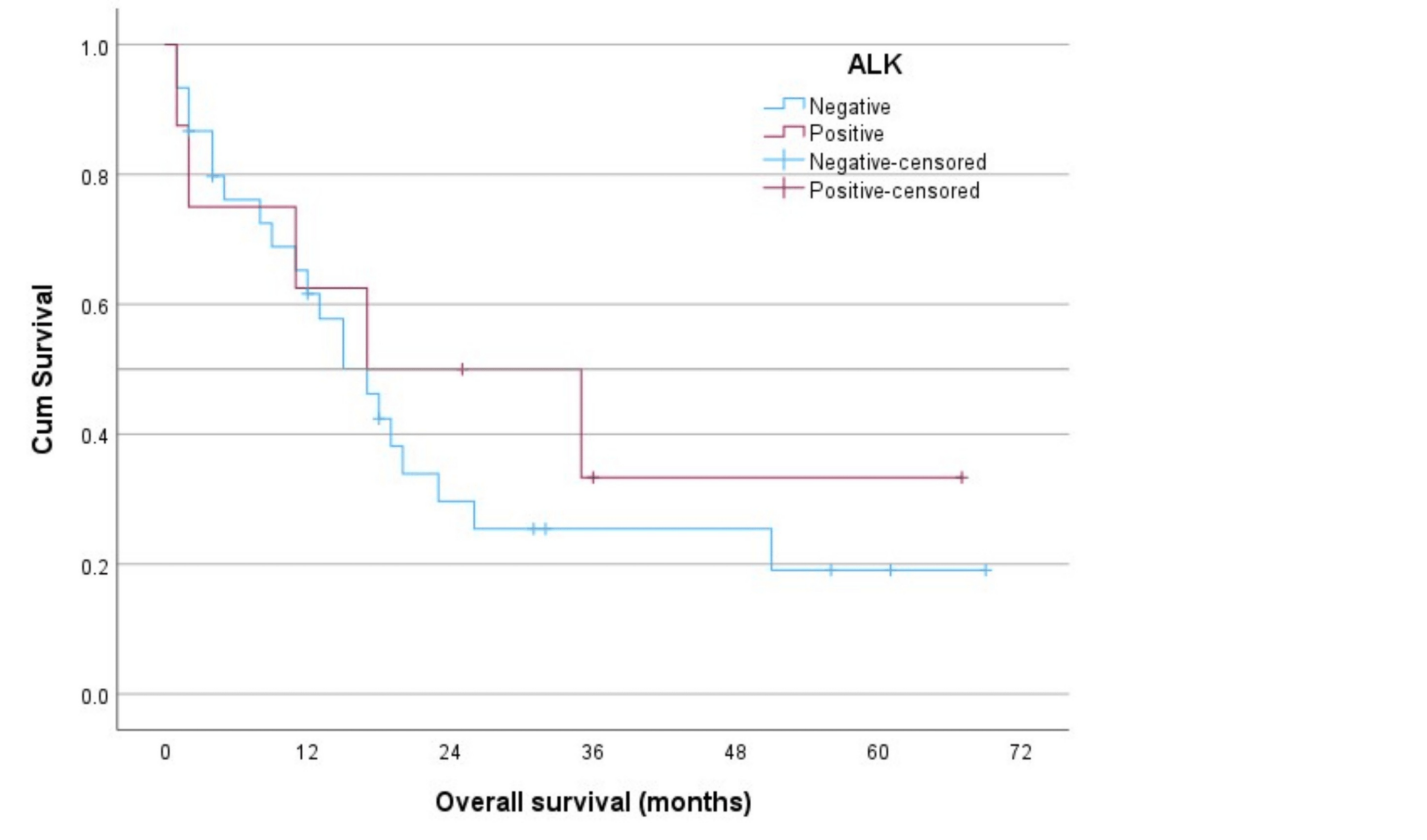
The survival distributions by the anaplastic lymphoma kinase (ALK) status in lung cancer patients using the Kaplan-Meier analysis.

The logistic regression did not show any significant associations with the outcome of interest (dead versus alive). For the Kaplan-Meier survival analysis, death was the final patient status. Figure 1 displays the survival distributions according to the EGFR status. Figure 2 displays these according to the ALK status.

The log-rank test (Mantel-Cox) comparing the overall equality of survival distributions by the EGFR status was not statistically significant (chi-square = 0.011, df = 1, and p-value = 0.916); neither was the comparison between the ALK status (chi-square = 0.414, df = 1, and p-value = 0.520). Table 2 shows the descriptive results for the EGFR status. Table 3 shows these for the ALK status.

**Table 2:** The results of the Kaplan-Meier analysis for lung cancer patients based on their epidermal growth factor receptor (EGFR) status.

Kaplan-Meier					Survival estimate (months)				
EGFR	Total N	Dead	Censored	%	Mean	Median	Std. error	CI: Lower bound	Upper bound
Negative	40	29	11	27.5	24.857	15	2.925	9.268	20.732
Positive	10	8	2	20	19.767	16	4.743	6.703	25.297
Overall	50	37	13	26	24.584	16	2.384	11.327	20.673

**Table 3:** The results of the Kaplan-Meier analysis for lung cancer patients based on their anaplastic lymphoma kinase (ALK) status.

Kaplan-Meier					Survival estimate (months)				
ALK	Total N	Dead	Censored	%	Mean	Median	Std. error	CI: Lower bound	Upper bound
Negative	30	21	9	30.0	25.232	17	3.104	10.917	23.083
Positive	8	5	3	37.5	32.042	17	14.546	0	45.511
Overall	38	26	12	31.6	26.78	17	2.852	11.409	22.591

The median survival time for the EGFR-negative patients was 15 months, as opposed to 16 months for those who were EGFR-positive. Likewise, the median survival time for both the ALK-negative and positive patients was 17 months each.

## Discussion

Lung cancer is the second most common cancer in men and the third most common cancer in both sexes combined in Pakistan [1]. NSCLC is the most common type with a high incidence of acquired somatic mutations [2]. In clinical practice, thorough profiling of clinically relevant mutations is essential in identifying the most effective targeted therapy and understanding drug resistance mechanisms [8,9].

Currently available treatments for NSCLC primarily consist of radiotherapy, chemotherapy, surgery, targeted therapy, and immunotherapy. Unfortunately, most patients are diagnosed with advanced lung cancer (57%), and the five-year relative survival rate for those with metastatic lung cancer is only 6% [10]. The prognosis for patients with NSCLC has significantly improved with the introduction of targeted therapies. Several clinical trials have demonstrated the efficacy of targeted therapy that has led to significantly longer progression-free survival (PFS) than those who receive chemotherapy regimens [11-13].

This study evaluated the clinical, molecular, and demographic characteristics of patients with NSCLC, with a focus on variables involving disease stage, survival rates, and mutations in the EGFR and ALK genes. The results provided important insights into the cohort of 51 patients and an in-depth understanding of the complexities associated with NSCLC management. Our study's demographic analysis showed 65.4% male predominance, which is in accordance with the worldwide trend [14]. The average age of 59.6 years at the time of diagnosis is also in line with worldwide data. Of the patients, 84.6% had advanced stage IV disease at the time they were initially diagnosed, highlighting the challenges in diagnosing NSCLC at earlier stages in countries like Pakistan. Unfortunately, this number is much higher than in the Western world due to a lack of screening modalities, access to healthcare facilities, and lack of education [15].

We also focused on EGFR and ALK mutations, which are important biomarkers in NSCLC to define indications of targeted therapies. Approximately 45-50% of Asian patients with lung adenocarcinoma have EGFR mutations and the prevalence of ALK mutations is around 7% [12,13].

Of the patients, 19.2% had EGFR mutations, which is in line with the available literature, especially in the Asian population with a higher incidence of adenocarcinoma [12,16,17].

EGFR-positive patients in our study had a median survival of 16 months, whereas EGFR-negative patients had a median survival of 15 months. The median survival for both ALK-positive and ALK-negative patients was 17 months. Since EGFR-positive and ALK rearrangements have generally been linked to better prognoses and responsiveness to targeted therapy, our results contradict expectations [18-20].

The study analyzed the impact of EGFR and ALK mutations on patient outcomes, revealing that although the p-values did not reach statistical significance (p = 0.084 for EGFR and p = 0.255 for ALK), the observed values and cumulative survival probabilities suggest the potential influence of these genetic alterations on NSCLC prognosis. The study emphasizes the need for larger cohorts in molecular studies to draw significant conclusions [21,22].

This study aligns with established trends in NSCLC demographics, highlighting male predilection and the challenge of diagnosing advanced-stage disease. Patients harboring EGFR mutations were treated with tyrosine kinase inhibitors (TKIs), such as osimertinib or erlotinib, while those with ALK rearrangements received ALK inhibitors, such as alectinib. The response to these targeted therapies was noted but did not achieve statistical significance within the study's sample size.

The complexity of NSCLC and treatment responses is evident in the mismatch between survival results and molecular state. Factors such as ALK rearrangements and EGFR mutations, patient-specific characteristics, genetic changes, treatment modalities, and tumor heterogeneity significantly impact the disease's progression and response to therapy, thereby affecting survival rates.

In the precision oncology era, understanding the complex molecular landscape of NSCLC is crucial for effective therapeutic management. Integrating molecular subtyping, genomic profiling, and treatment response monitoring is essential for maximizing patient outcomes and identifying novel therapeutic targets to overcome treatment resistance.

Strengths and limitations

This study offers critical insights into the clinical, molecular, and demographic aspects of NSCLC in Pakistan, describing key biomarkers such as EGFR and ALK mutations. By aligning with global data on age and male predominance, the findings highlight regional challenges, particularly the high prevalence of late-stage diagnoses due to limited healthcare access and lack of screening. Despite its contributions, the study's small sample size, and exclusion of other significant mutations, such as KRAS or ROS1, limit the strength of its conclusions. These limitations emphasize the need for larger, prospective studies incorporating comprehensive molecular profiling to advance understanding and improve outcomes in NSCLC.

## Conclusions

The study enhances our understanding of NSCLC and regional trends but acknowledges limitations due to molecular studies and smaller sample sizes. It highlights the potential impact of EGFR and ALK mutations on survival outcomes and suggests future prospective trials with larger cohorts and additional variables to address the complexities of NSCLC.
